# Gangliosides and Cholesterol: Dual Regulators of Neuronal Membrane Framework in Autism Spectrum Disorder

**DOI:** 10.3390/ijms26031322

**Published:** 2025-02-04

**Authors:** Borna Puljko, Marija Štracak, Svjetlana Kalanj-Bognar, Ivana Todorić Laidlaw, Kristina Mlinac-Jerkovic

**Affiliations:** 1 Laboratory for Molecular Neurobiology and Neurochemistry, Croatian Institute for Brain Research, School of Medicine, University of Zagreb, 10000 Zagreb, Croatia; borna.puljko@mef.hr (B.P.); svjetlana.kalanj.bognar@mef.hr (S.K.-B.); 2 Department of Chemistry and Biochemistry, School of Medicine, University of Zagreb, 10000 Zagreb, Croatia; 3 General Hospital “Ivo Pedišić”, 44000 Sisak, Croatia; mstracak@gmail.com; 4 Department for Forensic Psychiatry, University Psychiatric Hospital Vrapče, 10090 Zagreb, Croatia

**Keywords:** glycosphingolipids, lipidome, autistic disorder, sphingolipids, lipid rafts

## Abstract

Autism spectrum disorder (ASD) is a neurodevelopmental disorder with heterogeneous clinical presentation. Diagnosing ASD is complex, and the criteria for diagnosis, as well as the term ASD, have changed during the last decades. Diagnosis is made based on observation and accomplishment of specific diagnostic criteria, while a particular biomarker of ASD does not yet exist. However, studies universally report a disequilibrium in membrane lipid content, pointing to a unique neurolipid signature of ASD. This review sheds light on the possible role of cholesterol and gangliosides, complex membrane glycosphingolipids, in the development of ASD. In addition to maintaining membrane integrity, neuronal signaling, and synaptic plasticity, these lipids play a role in neurotransmitter release and calcium signaling. Evidence linking ASD to lipidome changes includes low cholesterol levels, unusual ganglioside levels, and unique metabolic profiles. ASD symptoms may be mitigated with therapeutic interventions targeting the lipid composition of membranes. However, restoring membrane equilibrium in the central nervous system remains a challenge. This review underscores the need for comprehensive research into lipid metabolism to uncover practical insights into ASD etiology and treatment as lipidomics emerges as a major area in ASD research.

## 1. Introduction

Autism spectrum disorder (ASD) represents a unifying term for neurodevelopmental disorders with heterogeneous clinical presentations manifesting in early developmental periods. ASD is marked by persistent deficits in social communication and social interaction as well as some type of restricted, repetitive patterns of behavior, interests, or activities [1,2]. Even though diagnosing ASD is becoming more straightforward, nevertheless, despite decades of research, it has been difficult to elucidate the underlying cause(s) and identify all the major contributing factors due to the overwhelming phenotypic diverseness of the disorder and lack of biomarkers with confirmatory values. The main limitation in the way we currently diagnose ASD is the need for painstaking and lengthy behavioral assessments that can delay early intervention. Studies investigating the underlying causes of and contributors to the development of ASD have mainly focused on transcriptomics and metabolomics [3,4,5], with lipidomics analyses only recently arising as having the potential to provide fresh input for understanding ASD. Considering that lipids constitute an astounding amount of both white and grey brain matter [6], studying lipids in the context of ASD holds the potential to deepen our understanding of the brain structure, function, and disrupted homeostasis in ASD. Neuronal membranes have long been an intriguing and attractive research target. With distinct lipid and protein composition, excitability, and signal transduction traits, aberrant neuronal membrane functioning has been linked to many disorders and pathological conditions. Membrane lipids have stood in the shadow of their protein partners for too long but have been proven as key biochemical drivers of the cell’s fate.

This narrative review explores how two key lipid components of membranes in the central nervous system, gangliosides and cholesterol, can serve as dual regulators of the neuronal membrane structure and function. Specifically, we explore the potential combined effect of cholesterol and complex membrane glycosphingolipids in the development of ASD, offering a more comprehensive approach to understanding ASD, since there are no studies investigating their joined impact on ASD pathophysiology. However, recent findings in lipid research reveal new plausible mechanisms of how the disturbed complex lipid and sterol metabolism can have a concerted effect resulting in different pathological cascades. Those discoveries, together with the insight from studies focusing on lipids in ASD, encompassing not only research performed on brain tissue but also studies reporting changes in ganglioside and cholesterol concentrations in the blood plasma of ASD subjects, can lead to establishing new, easily detectable biomarkers for the diagnosis of ASD. We propose that the changes in lipid profiles of cholesterol and gangliosides could aid in early diagnosis of the disorder, whilst interventions aimed at restoring lipid homeostasis may ameliorate ASD symptoms, providing additional options in early intervention. This work can, therefore, contribute to paving the road between basic lipidomics research and translational applications in ASD management.

## 2. The Intricacy of Diagnosing Autism Spectrum Disorder (ASD)

**Figure 1:**
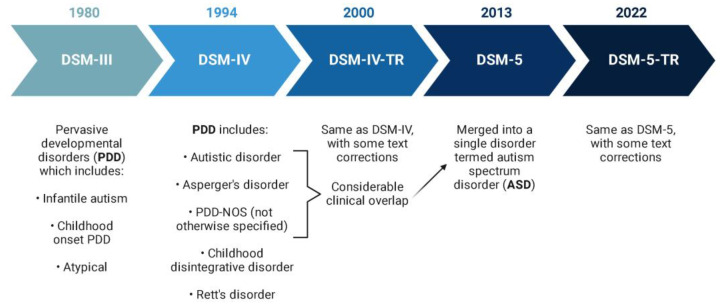
The overview of the evolution of the term autism spectrum disorder according to the Diagnostic and Statistical Manual of Mental Disorders (DSM) published by The American Psychiatric Association (APA) [1,2,11,12]. The figure was created with Biorender.com.

The onset of ASD is in infancy or early childhood. Inheritable and environmental factors influenced by epigenetics all play a major role in the etiology of ASD. The heritability of autism is estimated between 70 and 90% [7,8] and it affects about 2% of children with a 4:1 male-to-female ratio [9]. The accurate description of the autism category is complex. Clinical conditions can be recognized with good reliability, but we still do not have the skills to define them in a specific way. Signs and symptoms that characterize ASD do not always occur at the same time and they can also become noticeable and then attenuate [10]. The term autism as a heterogeneous disorder is used in various ways, and during the last century, it was part of the category of pervasive developmental disorders (PDD). This category was utilized as a term in the Diagnostic and Statistical Manual of Mental Disorders, Third Edition (DSM-III) in 1980 [11] to disseminate the idea of a wider spectrum of social communication deficits. In the DSM fourth edition (DSM-IV), pervasive developmental disorders included three disorders (i.e., autistic disorder, Asperger’s disorder, and PDD-not otherwise specified; PDD-NOS) with considerable clinical intersection, but also included other disorders and syndromes, namely childhood disintegrative disorder and Rett syndrome, as shown in Figure 1 [12,13]. The main disadvantage of the PDD category was the difficulty in reliably distinguishing the individual disorders. The most recent diagnostic systems, the International Classification of Diseases 11th Revision (ICD-11) [14] and DSM-5 as well as the DSM-5–Text Revision [1,2], use the umbrella term ‘ASD’ (autistic spectrum disorder) to identify individual differences using additional clinical specifiers and modifiers [15]. In the DSM-5, the DSM-IV’s separate PDD diagnoses (autistic disorder, Asperger’s disorder, and PDD-NOS) were combined into one, creating the ASD spectrum concept (Figure 1). Rett syndrome is considered an individual neurological disorder. A separate social (pragmatic) communication disorder (SPCD) was identified for those with impairments in social communication, but missing repetitive, restricted behaviors. The severity level descriptors were added to classify the level of support needed. This new definition was intended to be more accurate at diagnosing ASD at an earlier age and studies estimated that the impact of moving from the DSM-IV to the DSM-5 would see a decrease in ASD prevalence [16].

According to DSM-5, the essential features of ASD are persistent impairment in reciprocal social communication and social interaction (Criterion A), and restricted, repetitive patterns of behavior, interests, or activities (Criterion B). These symptoms are present from early childhood and limit or impair everyday functioning (Criteria C and D). The stage at which functional impairment becomes obvious will vary according to the characteristics of the individual and his or her environment. Core diagnostic features are evident in the developmental period, but intervention, compensation, and current support may mask difficulties in at least some contexts. Manifestations of the disorder also vary greatly depending on the severity of the autistic condition, developmental level, chronological age, and possibly sex; hence the term spectrum [2]. In addition to impairments in social communication and interaction, repetitive behaviors, and sensory anomalies with different levels of intellectual disability, autistic youngsters frequently experience a diapason of cognitive, learning, language, medical, emotional, and behavioral problems. These obstacles include a need for routine, difficulty understanding other people’s intentions, feelings, and perspectives, and sleeping and eating disturbances. Mental health problems, including anxiety, depression, attention disorders, self-harm behavior, and aggressive behavior, can also be present in people with autistic spectrum disorder [17]. Co-occurring psychiatric and neurological disorders such as hyperactivity and attention disorders (attention-deficit/hyperactivity disorder, ADHD), anxiety, depression, and epilepsy are prevalent in people with autism spectrum disorder [16]. In addition, language delays, motor problems, epilepsy, difficulties with sleep and eating, and high levels of activity are most frequently recognized in preschool children with autism. At the same time, ADHD, anxiety, obsessive-compulsive disorder (OCD), intellectual disability, academic challenges, irritability, and disruptive behaviors are commonly found in school-aged children [15]. Studies show that adults with autism are more likely to be diagnosed with many other physical health conditions, such as immune conditions, sleep disorders, and obesity than adults in the general population. Gastrointestinal disorders, including dietary restrictions and food selectivity, sleep disorders, obesity, and seizures are also common co-occurring disorders [16]. All these impairments can influence the quality of life of an individual, and their family or career, and make them socially vulnerable. The clinical manifestation of autism can differ depending on the severity of autism itself, the presence of coexisting conditions, and levels of intellectual ability. Intellectual disability can range from severe to average or above average intelligence quotient (IQ) [17]. All these comorbidities, together with the complex presentation of ASD, make it especially challenging to diagnose and elucidate the underlying causes.

At present, there is no clear biomarker of ASD, and the diagnosis is made based on observation and accomplishment of criteria described in DSM-5 and ICD-11. Considering the relatively high yield in patients with ASD, clinical genetic testing is recommended. It can give information concerning necessary medical interventions or workups and help with family planning. The American College of Medical Genetics and Genomics guidelines [18] currently recommend chromosomal microarrays for all children, fragile X testing in males, and additional gene sequencing in certain patients as first-tier genetic testing in the workup of ASD [16]. According to both DSM-5 and ICD11 criteria, the symptoms of ASD must be present before the age of three. A diagnosis is established after taking a detailed developmental history and observing the patient interacting with parents or other individuals [15]. The ADOS-2 (Autism Diagnostic Observation Schedule) and ADI-R (Autism Diagnostic Interview-Revised) are currently the gold standards for the diagnosis of ASD according to the international consensus. This acknowledgment does not mean that there are no other tools, which include the CARS (Childhood Autism Rating Scale), GARS (Gilliam Autism Rating Scale), DISCO (Diagnostic Interview for Social and Communication Disorders), or computerized tools such as 3DiA (Diagnostic Autism Rating Scale) [19].

The early detection of ASD allows intervention to be initiated even before a formal diagnosis is made, at a critical time in neurodevelopment, which consequently causes better outcomes and prognoses. Early preventive measures for children with ASD are crucial due to prevalent communication difficulties. They are carried out before the start of compulsory schooling, which, depending on the country and legislation, is usually established at around the age of six [19]. The types of measures vary throughout life and consist of parent-mediated interventions and/or therapist-delivered interventions in childhood and school-based strategies and techniques to promote independence in adulthood. Irritability, as an example of associated symptoms of ASD, as well as comorbidities such as anxiety can be treated with pharmacological therapies [16]. For early intervention to be possible, early detection is necessary [19], highlighting once more the dire need for a better understanding of the root causes of ASD so adequate and timely treatment can be introduced.

## 3. Lipidomics Research Toolbox

Following the massive advancements in scientific methodology, the lipidomics research toolbox has also diversified and become more sophisticated. Historically, gas chromatography (GC)-mass spectrometry (MS) has provided invaluable insights, being a major tool for lipid profiling, but today the spotlight is set on higher throughput methods with increased sensitivity and resolution power [20]. Two strategies can be utilized in lipidomic analysis: the global shotgun approach identifying and/or quantifying a maximum number of lipid compounds in preferably a small amount of samples, and targeted lipidomics, where each class of lipid is analyzed individually by extraction and detection procedures designed for that particular lipid class [21]. Each of these approaches has its own advantages. The shotgun approach can identify new metabolites associated with a particular condition, while targeted lipidomics can provide deeper insight into the connection between the chosen metabolite and the condition of interest. The technical implementation can vary and there are a variety of techniques being adopted for both approaches, where the most advanced techniques have already spurred many breakthroughs in the research of neurodevelopmental disorders. For example, the use of untargeted lipidomics has uncovered novel lipid signatures, such as oxidized phospholipids, which are linked to oxidative stress and neuroinflammation in ASD [22]. Tandem mass spectrometry (MS/MS) and ultrahigh-performance liquid chromatography-MS (UHPLC-MS) can detect minute metabolic alterations connected to ASD and provide high-resolution lipid species profiling [23,24]. For the first time, single-cell lipidomics has revealed cellular lipid heterogeneity in neuronal and glial cells connected to ASD pathogenesis [25,26]. Furthermore, imaging MS (IMS) has mapped brain tissue lipid distributions, illustrating how dysregulated lipid rafts (LRs) impact receptor clustering and synapse architecture [27]. With the latest advancements in lipidomics, researchers can now identify the lipid-gene-environment interaction in ASD by integrating these data with proteomics and transcriptomics. These developments, in conjunction with spatially resolved approaches such as nanoscale secondary ion mass spectrometry (NanoSIMS) and machine learning, could reveal hitherto unknown lipid dynamics [28].

Furthermore, there are more specific techniques available to study the particularities of different lipid classes, e.g., gangliosides. Various scattering techniques (laser light, neutron, and X-ray scattering), nuclear magnetic resonance (NMR), capillary electrophoresis-laser induced fluorescence, and immunoelectron microscopy, are just a few that generated results regarding the abundance, structural features, and behavior of these complex lipids [29,30,31,32]. The data generated by these techniques and approaches can be integrated with software and bioinformatics tools to uncover more complex interactions and effects [33] and can be complemented with the research on artificial membranes and molecular dynamics simulations [34,35].

Having all these methodologies at our disposal, what have we learned about lipid involvement in ASD?

## 4. Neurolipid Signature of ASD

Lipids are the most abundant components of the brain, comprising almost 80% of the dry weight of axon myelin sheaths and up to 40% of the neuron-rich gray matter [34]. It is not only a matter of quantity: the brain has a profoundly unique lipidome and the alterations in expression, micro-location, and metabolism of brain lipids can result in different pathological conditions [36]. The brain contains a staggering 23% of all cholesterol in the body [37], which is why cholesterol metabolism has been heavily investigated regarding many neurological diseases, especially since cholesterol cannot penetrate the blood-brain barrier, meaning that cholesterol metabolism in the brain is separate from the cholesterol metabolism in the rest of the body [38]. Gangliosides, complex membrane glycosphingolipids, are also major brain lipidome constituents. Glycosphingolipids represent around 80% of the total glycan mass in the brain, and gangliosides carry almost 75% of the brain’s sialic acid [39,40]. As such, both cholesterol and gangliosides have raised interest as likely having a role in the development of ASD. An overview of studies investigating either cholesterol or gangliosides in ASD subjects is given in Table 1 and Supplementary Table S1.

The findings overviewed in Table 1 and Supplementary Table S1 are mostly derived from patients’ and controls’ blood samples. The obvious difficulty in studying brain pathologies and disorders lies in the unavailability of human tissue, which is, with few exceptions, available only post-mortem. Still, valuable clues can be identified in studies performed in extraneural tissues and cells. For instance, some clinical features of ASD appear to be associated with the investigated biochemical parameters, e.g., hyperactivity level was greater when erythrocyte membrane fluidity was higher together with lower polyunsaturated fatty acid (PUFA) concentration in erythrocyte membranes [41]. The severity of hyperactivity is also found to be correlated to erythrocyte saturated fatty acids (SFA) and palmitic acid concentration [42,43,51]. Cholesterol is the main, bidirectional modulator of membrane fluidity and several studies uniformly report lower cholesterol concentrations (hypocholesterolemia) in ASD individuals compared to healthy children and teenagers [42,43,53]. Other studies have also shown aberrations in different lipid blood metabolites such as docosahexaenoic acid (DHA), and phenotypically distinct ASD groups can even be traced to specific metabolomics profiles [47]. However, the opposite direction of the observed effect is reported, and some of the ASD subgroups, according to their metabolomics profile, have decreased lipid metabolism, while other subgroups have increased metabolism of membrane lipids and fatty acid byproducts [41]. Conflicting data are also reported for the sialic acid and/or ganglioside concentration in the blood plasma of ASD subjects. Some studies did not detect differences in sialic acid content between groups [44,45,46,47,50], while other studies report significant differences in concentration of sphingosine 1-phosphate, sialic acid, ceramide, sphingomyelin, sphingosine, glucosylceramides, gangliosides, and higher flux of sphingolipid metabolism in general [44,45,46,47,52]. Cerebrospinal fluid (CSF) reveals significant perturbations in ganglioside presence in ASD, where ganglioside GM1 seems to be particularly prominent with an elevated concentration in ASD (Table 1 and Supplementary Table S1) [45,46,52,53]. In addition, serum levels of anti-ganglioside M1 antibodies are found to be significantly higher in ASD than in healthy children [45,46,54,55], even though there are studies that did not find the association between anti-ganglioside antibodies and ASD [56,57].

Even though some of these studies produce seemingly conflicting data, it is indisputable that they all suggest a disequilibrium in membrane lipid content. The contradictions observed in sphingolipid levels in the reviewed studies could have arisen from several factors. Sphingolipid metabolism is complex and some of the metabolites reported in these studies (Supplementary Table S1 and Figure S1) participate in processes serving opposite roles, e.g., ceramide and ceramide-1-phosphate, or sphingosine and sphingosine-1-phosphate, which can be quite responsive to environmental factors [58,59]. Furthermore, the differences in fatty acid (FA) content can be due to not only the FAs within sphingolipids (Supplementary Figure S1) but also FAs released from phospholipids, which were not the focus of this review. Moreover, the composition and concentration of gangliosides will significantly change during neurodevelopment and aging, therefore yielding completely different patterns at different stages in life [60,61]. The studies reviewed in this work encompassed subjects of different mean ages, and the response of specific sphingolipid species towards extracellular and cell signals may change differently during the development of a specific disorder, therefore producing seemingly opposite results. The studies reporting conflicting results were performed on a modest number of subjects, the largest one encompassing less than 200 ASD cases and 200 controls (Table 1). The only larger study performed on 2000 ASD individuals did not have a control group since the aim was to derive phenotypically driven subgroups of ASD and investigate their respective metabolomes [48,49]. Since ASD affects about 2% of children [48], larger studies are needed to draw more confident conclusions. On the other hand, ASD affects males much more than females, which is well-represented in the study populations (Supplementary Table S1). The methodologies used in these studies are also quite heterogeneous. The levels of sialic acid are customarily assessed by colorimetric methods, which lack the sensitivity of MS methods. The same is valid for ganglioside levels detected in CSF using the microimmunoaffinity technique (Supplementary Table S1), which can only detect the major brain gangliosides [47,50], therefore completely omitting sphingoid bases, other gangliosides, and sphingolipids in general from the analysis. Hence, this targeted approach uniformly detected elevated ganglioside concentrations in CSF; however, the analysis did not encompass the sphingolipid metabolites reported by other untargeted analyses. Nevertheless, altered central nervous system (CNS) ganglioside patterns detected in CSF may reflect important correlates of pathogenic events taking place in ASD [47,52]. Brain transcriptome data and metabolic modeling, together with metabolomics profiling of large patient cohorts [58], generated the most data regarding specific sphingolipids, and the results of those studies point to a disturbance of overall membrane lipid fine-tuning closely related to a specific subgroup of ASD phenotypes. This is especially noteworthy for two reasons: (1) the reviewed studies presented in Table 1 have been conducted at different times and therefore the diagnosis was given according to different diagnostic criteria, which inherently results in discrepancies in the results; (2) despite the enormous heterogeneity of ASD presentation and severity, ASD subjects are still often analyzed as a single uniform group. However, when a larger number of ASD subjects is divided into subgroups according to their phenotypes [60], distinct sphingolipid profiles emerge. Undoubtedly, grouping ASD individuals into more phenotypically homogeneous groups according to the severity and clinical symptoms would result in more interpretable straightforward results [62]. In summary, future research may overcome these apparent disparities by standardizing lipid extraction and evaluation, splitting participants by age, sex, and ASD severity, and tracking lipid profile changes longitudinally [62].

The conclusion that we can draw from these studies points to a scenario where hypocholesterolemia regularly accompanies ASD. We can imagine it sets the stage for membrane disturbances and facilitates additional aberrations in gangliosides (and other sphingolipids) metabolism, which will lead to a pathogenic cascade, even though aberrations in ganglioside metabolism can be the drivers of membrane-wide cholesterol redistribution. The malfunctioning and disturbed neuronal connectivity seen in ASD may be caused by these membrane-related changes, which emphasizes the significance of comprehending the composition and structure of neuronal membranes in both normal and abnormal neurodevelopment [63]. Thus, what are the possible downstream effects of altered neural membrane lipidome?

## 5. Primary Rendezvous Point of Gangliosides and Cholesterol

Membranes are by no means uniform in their entirety. They are highly dynamic structures composed of distinct microdomains with particular lipid and protein composition. Specialized microdomains termed lipid rafts, especially abundant in cholesterol and (glyco)sphingolipids, are essential for numerous cellular processes [64]. LRs can be considered as organizing centers for the cell, promoting the aggregation of receptors and signaling molecules, hence improving the efficacy of cellular communication [65,66]. In neurons, they play a pivotal role in the organization of neurotransmitter receptors, ion channels, and signaling proteins, thereby being crucial for processes such as synaptic transmission, plasticity, and neuronal growth [67]. Dysregulation of lipid raft dynamics can lead to impaired signaling and synaptic dysfunction, which are often observed in ASD [68,69]. Research has demonstrated modifications in cholesterol metabolism and lipid raft composition in ASD models, including Fragile X syndrome [70,71]. These alterations are linked to atypical transmembrane signaling, synaptic impairments, and clinical symptoms of ASD [72,73,74].

**Figure 2:**
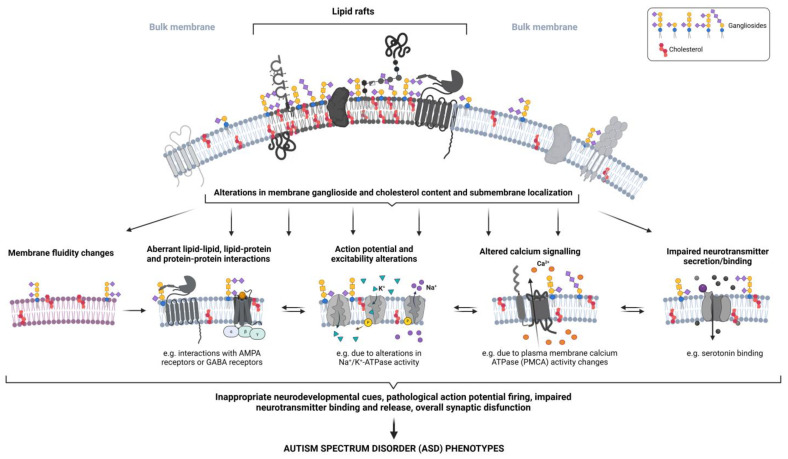
The overview of possible consequences of altered membrane ganglioside/cholesterol content on disturbed function of neural membranes. The figure was created with Biorender.com.

As already stated, the main lipid LR components are cholesterol and glycosphingolipids [75]. The association between cholesterol and ASD is intricate, encompassing genetic influences, lipid raft functionality, and inflammatory mechanisms [73]. Gangliosides are also intimately linked to neurodevelopment, synaptic plasticity, and neuronal function [44,45,46,47,48,49,52,54,55]. We already highlighted aberrant ganglioside concentrations as well as levels of sialic acid, sphingosine, and ceramide, the building blocks of gangliosides, in ASD [40,74,75,76,77,78,79]. Gangliosides are present in the outer leaflet of the plasma membrane, with their hydrophobic ceramide anchor immersed in the lipid bilayer, and their complex glycan chain marked with the presence of at least one molecule of sialic acid protruding into the extracellular space (Supplementary Figure S1). As such, they are highly versatile in their interactions, which can be achieved through both *cis*, laterally within the same membrane, and *trans* associations, with extracellular matrix and different types of ligands. Therefore, gangliosides can maintain an array of molecular associations in lipid rafts, and the disruption of their metabolism can lead to developmental defects as well as neurological and neurodegenerative pathologies [40,76,77,78,79,80,81] (Figure 2). However, we cannot observe cholesterol perturbations and ganglioside perturbations separately; it is their cumulative or concerted effect that could lead to detrimental effects.

## 6. Disturbances in Ganglioside-Cholesterol Synergy May Lead to Biochemical Imbalances in ASD

Changes in neuronal membranes, including lipid profiles, receptor kinetics, membrane fluidity, and cytoskeletal interactions, affect the ASD neurobiology [36,82,83,84]. Neuronal membrane lipid composition is essential for healthy synaptic plasticity and function, both of which are frequently compromised in this disorder [85]. Cholesterol and gangliosides can jointly modulate the fluidity of the membrane, which is crucial for the proper functioning of membrane proteins; cholesterol tends to stabilize membrane structure, while gangliosides introduce specific interactions that affect fluidity [74,86]. This section gives insights into the possible effects of ganglioside-cholesterol combined nexus on the known biochemical imbalances observed in ASD. The interplay between cholesterol and gangliosides in lipid rafts may provide a unifying mechanism and the basis for the influence exerted by diverse genetic and environmental risk factors associated with ASD (Figure 2).

### 6.1. Molecular Mechanisms and Mechanistic Insights into the Influence of Gangliosides and Cholesterol on Membrane Proteins

Numerous invaluable fundamental biophysical studies have been conducted to elucidate the exact molecular mechanisms of how particular membrane lipids interact with each other and with membrane proteins. Approaches investigating biomimetic membranes with controlled lipid composition combined with nanoscale experimental techniques have been extensively used for this purpose [87,88]. We highlight some of the examples in this section.

Various membrane systems can be established as biomimetic structures to study membrane mechanics: freely suspended and supported liposomes, giant unilamellar vesicles, micelles, lipid films (Langmuir monolayers, phospholipid bilayers nanodiscs, protein-tethered bilayer lipid membranes, hybrid bilayers), etc. [88]. The nanomechanics of these membrane systems can be characterized by complementary techniques such as quartz crystal microbalance with dissipation monitoring (QCM-D) and atomic force microscopy (AFM). Such studies characterized various membrane biophysical properties and provided the basis for the elucidation of many membrane parameters, e.g., mechanical properties, hydrophobic/hydrophilic interactions, and fusogenic properties [89,90].

**Figure 3:**
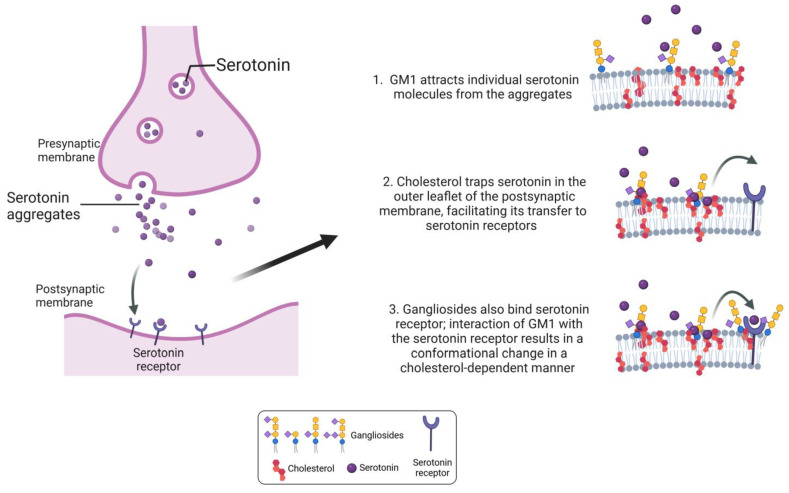
Schematic representation of the concerted effects of ganglioside GM1 and cholesterol on serotonin, illustrating a direct role of ganglioside-cholesterol interaction in modulating ligand binding and receptor function. The figure was created with Biorender.com.

In addition to intrinsic structural membrane properties, which provide the basis for all other membrane-related events, we are specifically interested in ganglioside-cholesterol-protein interactions, which were in part elucidated by in silico studies of reconstructed membrane environments [35,91]. For example, microtensiometry revealed the mode of interaction of ganglioside GM1 and a myelin sheath proteolipid plasmolipin (PLLP) together with cholesterol. GM1 induces the structuring of the extracellular loops of PLLP that propagates a conformational signal through the plasma membrane, involving a cholesterol molecule located between transmembrane domains of PLLP. This conformational wave is transmitted to the intracellular domain of the protein, conveying the signal intracellularly. This study is a very illustrative example of the chaperone effect of gangliosides on disordered protein motifs in lipid raft proteins [91]. This is by no means the only study investigating the effect of gangliosides on cholesterol-interacting proteins, as well as on cholesterol. Another example, also elucidated in model lipid membranes by a combination of in silico and physicochemical approaches, is the effect on serotonin [92]. Specifically, it is shown that serotonin aggregates are dissolved by postsynaptic membrane gangliosides, particularly GM1 (Figure 3). This is initially achieved through electrostatic forces, followed by a set of CH-π and van der Waals interactions between serotonin and cholesterol. By attracting individual serotonin molecules from the aggregates secreted in the synaptic cleft as a first step, gangliosides and cholesterol act together as a functional serotonin-collecting funnel on neuronal membranes. Cholesterol then traps serotonin in the outer leaflet of the postsynaptic membrane, facilitating its transfer to serotonin receptors [93], where gangliosides also bind serotonin receptors [94]. Interaction of GM1 with the serotonin receptor results in a conformational change in a cholesterol-dependent manner, which illustrates a direct role of ganglioside-cholesterol interaction in modulating ligand binding and receptor function, schematically represented in Figure 3 [95,96,97,98]. Many more studies using similar approaches revealed the copiousness in ganglioside-cholesterol-protein interactions within the membrane, detailly elaborating them [99]. Gangliosides can adopt different topologies, e.g., chalice-like at the edge of lipid rafts and condensed clusters in central raft areas. This is a consequence of their ability to neutralize the negative charge of the carboxylate group of sialic acids by the amide groups of N-acetylated sugars, the so-called “NH trick”, which can heavily impact protein binding [35,99]. Several types of ganglioside-binding domains are known and the modes of interaction of those domains are described with proteins such as synaptotagim, serotonin receptor, β-amyloid peptide, cholera, and botulinum toxins, etc [96,97]. Examining these interactions is in line with ganglioside versatile structure and the mentioned metamorphism possibilities, where they can dictate and modulate the geometry and the topology of the membrane along with its mechanical properties [97,98].

Another experimental approach using clickable photoaffinity ganglioside probes revealed a rich human ganglioside interactome [99,100]. Major ganglioside interactome protein families include transmembrane transporters, cell adhesion molecule binding proteins, and cell regulatory proteins, including those of the receptor tyrosine kinase family, etc. Some ganglioside–protein interactions are selective for the precise ganglioside glycan structure, whereas other proteins interact with gangliosides in general, regardless of their glycan sequence [100,101].

To further accentuate the role of gangliosides in membrane organization and their consequent effect on membrane receptors’ function, studies report that cholesterol presence in the membrane is influenced by gangliosides. Specifically, membrane segregation of GM1 ganglioside can force the transbilayer distribution of cholesterol, which adds a new dimension to ganglioside-driven membrane organization [101]. It has been experimentally shown that gangliosides, specifically GM1, can push cholesterol to distribute largely into the cytosolic face of the membrane, which means that the presence of GM1 enforces an asymmetry in cholesterol distribution [101]. Furthermore, preferential asymmetric distribution of GM1 and cholesterol is attained in a model membrane with biomimetic composition, revealing that a true coupling between the two molecular species occurs [99]. This is supported by other research efforts describing the “lipid shape” concept to model the interactions between cholesterol and gangliosides. The basis of the concept lies in geometric complementarity between cholesterol and sphingolipids, where cholesterol fits in a cavity formed by ceramide and the glycan part of the ganglioside, and without cholesterol gangliosides would adopt a micellar organization, completely changing membrane structure [102].

Many molecular contacts exist through which gangliosides and cholesterol can affect each other, as well as exert a deep impact on membrane proteins and receptors. Hence, these findings offer a mechanistic rationale for the cholesterol-ganglioside synergy in the pathophysiology of ASD.

### 6.2. Key Roles of Both Gangliosides and Cholesterol in Neurodevelopment

Studies show that ASD arises from modified neurodevelopmental pathways that affect cortical connection, neuronal patterning, and brain growth. According to the “developmental disconnection” theory, higher-order association areas and the frontal lobe may partially separate throughout development [103]. A growth dysregulation hypothesis has been proposed to explain the neurobiological basis of ASD [104,105]. Both gangliosides and cholesterol have a profound impact on neurodevelopmental processes. Significant alterations occur to ganglioside levels throughout brain development. More complex gangliosides become prevalent later in development, and simpler gangliosides like GM3 and GD3 predominate in early embryonic stages [105]. In general, ganglioside concentration rises during the early stages of pregnancy and early childhood before gradually declining as people age [105]. They play a critical role in neurodevelopment, including supporting synapse formation and neural circuit development, neuronal growth, migration, myelination, and synaptogenesis, and disruption of ganglioside metabolism may contribute to neurological disorders [106]. They regulate axonal development by modulating membrane receptors and signaling pathways [107,108]. Supplementing with dietary gangliosides during pregnancy and the early postnatal period may have a beneficial effect on the development of the offspring’s brain and cognitive abilities [37,109]. The development and function of the brain also depend heavily on cholesterol, especially in the early stages of growth when the central nervous system demands high rates of cholesterol production, with it being essential for neuronal differentiation, synaptogenesis, and myelination processes [110].

### 6.3. Membrane Excitability and Membrane Lipids

Research on particular subtypes of prefrontal cortical neurons in ASD mouse models has revealed abnormalities in action potential generation and backpropagation [111]. Studies have demonstrated higher Na^+^/K^+^-ATPase activity in ASD persons’ frontal cortex and cerebellum [41,112], whereas decreased activity was identified in ASD children’s erythrocytes [112,113,114,115]. Interestingly, the same study showing decreased Na^+^/K^+^-ATPase activity in erythrocytes also detected altered membrane fluidity [113,114,115,116]. Na^+^/K^+^-ATPase is essential for setting resting membrane potential and restoring transmembrane Na^+^ and K^+^ gradients after neuronal firing, i.e., essential for stabilizing the cell membrane potential. Na^+^/K^+^-ATPase is biphasically affected by cholesterol; its activity is inhibited by cholesterol at both high and low concentrations [117,118]. Not only that, Na^+^/K^+^-ATPase also requires a particular ganglioside environment for optimal function [93,98,118], and it seems that it even directly binds certain ganglioside species, making Na^+^/K^+^-ATPase a part of ganglioside cellular interactome [94,99,119]. Therefore, even subtle transient perturbations in cholesterol/ganglioside membrane content could have an impact on neuronal excitability by directly affecting the function of key membrane ion transporters.

### 6.4. Signal Transducing Effects of Membrane Receptors Depend on the Membrane Lipid Composition

Both cholesterol and gangliosides can affect the trafficking and localization of membrane receptors [119,120]. Proper localization is crucial for receptor function, and disruptions in receptor function contributes to altered signaling pathways implicated in ASD [120,121]. One example is AMPA receptors (AMPAR). AMPAR trafficking and function have also been shown to be disturbed in a variety of ASD models, including genetic mutations and environmental variables [122]. Abnormalities in AMPAR expression, density, and signaling have been found in postmortem brain tissue derived from individuals with ASD [123,124]. Animal models of autism demonstrate altered glutamatergic transmission, with some having decreased and others having enhanced AMPAR activity [124]. Importantly, pharmaceutical manipulation of AMPARs has shown promise in improving social deficits in ASD animal models [125]. Cholesterol is required for AMPA receptor integrity and depletion of cholesterol/sphingolipids leads to instability of surface AMPA receptors and gradual loss of synapses (both inhibitory and excitatory) and dendritic spines [126]. In addition, gangliosides, particularly GT1b and GM1, play an important role in AMPAR trafficking [127,128,129,130,131,132].

Apart from AMPAR, the “wrong” combination of cholesterol and gangliosides can influence various signal transduction pathways, including those related to growth factors and neurotrophic factors [133]. Alterations in these pathways may contribute to the neurodevelopmental aspects of autism.

### 6.5. The Connection Between Neurotransmitter Release and Membrane Lipids

Several neurotransmitter systems are altered in ASD, including serotonin, dopamine, glutamate, and GABA [92,94,134,135,136]. In neurons, gangliosides and cholesterol act jointly to enhance the release of neurotransmitters by modulating the functions of synaptic proteins, as well as act as co-receptors in a chaperone-like capacity to promote the binding of the neurotransmitter to its receptor [94]. Moreover, typical binding sites allow cholesterol and sphingolipids to interact with neurotransmitter receptors, controlling their conformation and activity [137]. Presynaptic protein kinase activity, which controls neurotransmitter release, is also influenced by the amount of cholesterol in the membrane [138,139]. Depletion of cholesterol affects serotonin1A receptor activity and dynamics, whereas acute depletion inhibits synaptic transmission and plasticity in the hippocampus [140]. Neuroactive hormones and other stimulators also modulate GABAA receptors in response to membrane cholesterol levels [141]. As seen with adenosine 2A and dopamine D3 receptors, cholesterol affects G protein-coupled receptors (GPCR) heterodimerization [93].

Gangliosides, on the other hand, can interact with the serotonin1A receptor through the sphingolipid binding domain [142] and increase the affinity and potency of the receptors [143]. They lessen receptor supersensitivity and mitigate 6-OHDA-induced dopamine depletion in the dopaminergic system [144,145].

### 6.6. Cholesterol and Gangliosides Modulate Ca^2+^ Signaling

Recent studies indicate that calcium signaling disruption plays a crucial role in ASD. Inositol trisphosphate (IP3)-mediated calcium release from the endoplasmic reticulum has been reported to be diminished in both rare monogenic syndromes and sporadic ASD cases [145]. This calcium signaling deficiency appears to be a convergent point throughout the autism spectrum, with the potential to serve as a biomarker for diagnosis and therapeutic development [146,147]. Mutations in calcium channels and calcium-regulated proteins have been linked to ASD, emphasizing the significance of calcium-dependent gene expression in autism [134,148]. Cholesterol can influence calcium channel function, while gangliosides may modulate calcium signaling pathways [149], and the interplay between these lipids may contribute to dysregulation in ASD. They jointly provide an optimal membrane ecosystem, facilitating the proper functioning of these proteins.

Plasma membrane Ca^2+^-ATPase (PMCA) is critical for maintaining intracellular calcium homeostasis, especially in neurons [150]. Researchers have linked PMCA failure to a variety of neurological and neuropsychiatric conditions [151]. Specifically, ATP2B2 gene variations that encode one of the PMCA isoforms, PMCA2, have been linked to ASD in male patients [152]. Gangliosides and cholesterol have important roles in PMCA activity and location in neuronal membranes. Cholesterol stabilizes PMCA in lipid rafts, while gangliosides modulate its activity by direct binding [153]. Furthermore, cholesterol deprivation reduces raft-associated PMCA activity without altering the non-raft pool [154]. Gangliosides, notably GM1, regulate PMCA activity, with poly-sialogangliosides stimulating and mono-sialogangliosides inhibiting this ATPase [155]. PMCA4 is found only in lipid rafts and cholesterol/sphingomyelin-rich membrane regions, which may influence its interaction with Ca^2+^ signaling proteins [156]. GM1-containing lipid rafts stabilize PMCA-neuroplastin complexes, influencing calcium homeostasis in neurons [157,158,159,160]. Interestingly, neuroplastin, a synaptic glycoprotein that is, among its other diverse roles, an essential auxiliary subunit of PMCA, is investigated as a potential contributor to ASD [161]. Neuroplastin is also severely affected by its ganglioside environment [162]. Hence, there is a recurring theme that multiple components of different membrane complexes are all, separately or in tandem, influenced by either gangliosides, cholesterol, or both.

## 7. Lipid-Directed Treatment of Neurodevelopmental Disorders and Future Perspectives

The documented disequilibrium in membrane ganglioside/cholesterol content may suggest possible therapeutic interventions in neurodevelopmental and neuropsychiatric disorders. Specifically tailored supplementation options could improve some of the symptoms associated with ASD. GM1 treatment was found to improve the ASD model rats’ behavior disorders, including locomotor activity and exploratory behavior, social interaction, learning and memory capacity, as well as repetitive behavior [107,108]. Supplementing with dietary gangliosides during pregnancy and the early postnatal period also showed potential beneficial effects on brain development [163]. Cholesterol supplementation has also demonstrated some potential in treatment. Specifically, individuals with Smith–Lemli–Opitz syndrome, who universally show an autistic clinical phenotype [164], display fewer autistic behaviors and symptoms of irritability and hyperactivity, with improvements in physical growth, sleep, and social interactions after cholesterol supplementation. Other symptoms shown to improve with cholesterol supplementation include aggressive behaviors, self-injury, temper outbursts, and trichotillomania [165].

Though membrane lipid replacement therapy [165] is intermittently raised as a possibility, the question remains whether we can restore the membrane equilibrium in the CNS after any supplementation. However, we need to consider cholesterol and gangliosides in a more integrated manner. Specifically, we need to not investigate them separately but to consider the fatty acid composition in the ganglioside ceramide portion to obtain a more insightful view into the processes they influence. Gangliosides and cholesterol provide an optimal framework, allowing specific membrane proteins to function properly. There is also a matter of influence on downstream cellular events, again emphasizing the relevance and urgency of implementing lipidomics investigations, including their integration with genomics, epigenomics, proteomics, and multi-omics platforms to fully understand the pathophysiology of ASD and better explain and unify its wide array of symptoms and presentations [166]. The closely-knit network of interactions between gangliosides and cholesterol needs to be finely tuned, as the heterogeneous clinical phenotypes of ASD may, in part, stem from differential neural membrane compositions. To conclude, more comprehensive research efforts are necessary to uncover practical insights into ASD etiology and treatment as lipidomics emerges as a major area in ASD research.

## Figures and Tables

**Table 1:** The overview of studies investigating cholesterol and glycosphingolipids in ASD.

Sample Type	Subjects	Diagnosis	Glycosphingolipid/Cholesterol- Related Finding	Reference
Erythrocytes	41 children (21 ASD, 20 CON)	PDD/PDD-NOS according to DSM-IV-TR; autism according to ADOS	Erythrocyte membrane fluidity ↓ Monosaturated FAs content ↑ EPA (ω3) ↓ DHA (ω3) ↓ Membrane sialic acid content n.d.	[41]
Blood plasma/serum	100 children (83 autism, 14 BS, 3 NQA)	ADI-R derived three affected status categories: autism, NQA and BS	Cholesterol ↓	[42]
	158 individuals (79 ASD, 79 CON)	ASD according to DSM-IV or DSM-5 (dependent on diagnosis date)	Cholesterol ↓	[43]
	336 children (173 ASD, 163 CON)	Autism according to DSM-IV	Sphingosine-1-phosphate ↑ DHA ↓	[44]
	142 children (82 ASD, 60 CON)	ASD according to DSM-5, ADI-R and ADOS	Sialic acid ↓	[45]
	200 children (100 ASD, 100 CON)	ASD according to DSM-5	Sialic acid ↑	[46]
	2001 ASD	ASD according to ADI-R and ADOS; global metabolomics profiling revealed 3 phenotypically distinct subgroups *	Subgroup 1: sphinganine ↑; sphingosine ↑; sphingomyelins ↓ Subgroup 2: n.d. Subgroup 3: sphinganine ↓; sphingosine ↓; sphinganine-1-phosphate ↑; sphingosine-1-phosphate ↑; sphingomyelins ↑; ceramides ↑	[47]
Cerebrospinal fluid	114 children (85 ASD, 29 CON)	According to DSM-III	Ganglioside GM1 ↑	[48]
	45 children (20 ASD, 25 CON)	PDD according to ICD 10 classification	Gangliosides GM1, GD1a, GD1b and GT1b ↑	[49]
Postmortem prefrontal cortex transcriptome data [50,51]	60 individuals (29 ASD, 31 CON)	Autistic disorder according to ADI-R and ADOS	EPA ↓ DHA ↓ Sphingolipid metabolism flux ↑ Sphingosine-1-phosphate ↑ Ceramide ↓ Glucosylceramide ↑	[52]

The terms autism, ASD, autistic disorder, etc. are used as in the corresponding references. Age is given in years. ASD = autism spectrum disorder; CON = healthy controls; PDD = pervasive developmental disorder; PDD-NOS = pervasive developmental disorder–not otherwise specified; DSM = diagnostic and statistical manual of mental disorders; ADOS = autism diagnostic observation schedule; ADI-R = autism diagnostic interview-revised; L BS = broad spectrum; NQA = not quite autism; FA = fatty acid. EPA = eicosapentaenoic acid; DHA = docohexaenoic acid; n.d. = no difference; ↑ = elevated content in ASD compared to controls; ↓ = decreased content in ASD compared to controls. * Subgroup 1, children with the least maladaptive behavioral traits (N = 862); subgroup 2, children with the highest degree of challenges across all phenotype domains (N = 631); subgroup 3, children with maladaptive behaviors and co-occurring conditions that showed the highest IQ scores (N = 508).

## Supplementary Materials

The following supporting information can be downloaded at: https://www.mdpi.com/article/10.3390/ijms26031322/s1.
