# Supplementary material for: Gangliosides and Cholesterol: Dual Regulators of Neuronal Membrane Framework in Autism Spectrum Disorder
Source: Int J Mol Sci. 2025 Feb 4;26(3):1322. doi: 10.3390/ijms26031322 (PMC11818915; doi:10.3390/ijms26031322)
Supplement: Supplementary file 1 [file ijms-26-01322-s001.zip › ijms-3415320-supplementary.pdf]

Supplementary material for

Gangliosides and cholesterol: dual regulators of neuronal membrane framework in autism spectrum disorder

Borna Puljko [1,2†], Marija Štracak [3†], Svjetlana Kalanj Bognar [1,2], Ivana Todorić Laidlaw [4*] and Kristina Mlinac-Jerkovic [1,2*]

[1] Laboratory for Molecular Neurobiology and Neurochemistry, Croatian Institute for Brain Research, School of Medicine, University of Zagreb, 10000 Zagreb, Croatia;
[2] Department of Chemistry and Biochemistry, School of Medicine, University of Zagreb, 10000 Zagreb, Croatia;
[3] General Hospital "Ivo Pedišić", 44000 Sisak, Croatia;
[4] Department for Forensic Psychiatry, University Psychiatric Hospital Vrapče, 10090 Zagreb, Croatia
* Correspondence: kristina.mlinac.jerkovic@mef.hr (KM-J); fadopedrorodrigues@gmail.com (ITL)
† These authors contributed equally to this work.

Supplementary Table 1. Detailed comparison of methodological approaches between studies reviewed in Table 1 in the main text.

Sample type	Participants characteristics		Analyzed metabolites*	Methods for detection and analysis	Key findings	Notes	Ref.
	**Control** subjects number/ mean age/ sex	**ASD** subjects number/ mean age/ sex					
Erythrocytes	20 / 7.6 / 4 F, 17 M	21 / 6.8 / 4 F, 17 M	Sialic acid Fatty acids	Fluorescence anisotropy for membrane fluidity; periodate thiobarbituric acid method for sialic acid (colorimetry); gas chromatography for membrane lipids	Reduction of erythrocyte membrane fluidity, alteration in erythrocyte fatty acid membrane profile, no change in membrane sialic acid content	Hyperactivity score appears to be related with some parameters of the lipidomic profile and membrane fluidity	[41]
Blood plasma/ serum	No control subjects (results compared to reference values: CDC's National Health and Nutrition Examination Survey	100 / 9.5 / 22 F, 78 M	Cholesterol and all post-squalene sterol precursors	Dual column gas chromatography (flame ionization detector) FID + mass spectrometry	Up to 20% of children from a sample of mostly multiplex ASD sibships have substantial hypocholesterolemia (total cholesterol levels lower than 100 mg/dl, which is below the 5th centile for children over age 2 years)	Blood samples from a cohort of subjects with ASD from families in which more than one individual had ASD; samples were obtained from the Autism Genetic Resource Exchange (AGRE) specimen repository	[42]

Blood plasma/ Serum	79 /<br>19.4 /<br>15 F, 64 M	79 /<br>19.4 /<br>15 F, 64 M	Fasting lipid profile including total cholesterol, HDL-cholesterol, and triglycerides	The study examined preexisting medical records, no details are described regarding the analysis of the original data	Hypocholesterolemia more than threefold higher in ASD	Medical records from individuals with ASD were retrospectively reviewed; the ASD group was matched with a control group adjusted for age and gender which consisted of outpatients in the same institution	[43]
	Discovery cohort: 63 /<br>4.1 /<br>12 F, 51 M<br>Validation cohort: 100 /<br>4.5 /<br>19 F, 81 M	Discovery cohort: 73 /<br>4.6 /<br>14 F, 59 M<br>Validation cohort: 100 /<br>4.4 /<br>14 F, 86 M	The first cohort was used for a metabolomic discovery analysis of serum to identify metabolites that could contribute to the discrimination of cases and controls; suggested metabolites were then validated in an independent cohort of cases and controls	Liquid chromatography quadrupole time-of-flight tandem mass spectrometry (UPLC/Q-TOF MS/MS) in both positive and negative electrospray ionization (ESI) modes	Multiple logistic regression analysis identified sphingosine 1-phosphate and docosahexaenoic acid as significant predictors of autism	2-stage design with a discovery cohort of ASD cases and controls and an independent cohort of cases and controls for validation	[44]
	60 /<br>4.52 /<br>13 F, 47 M	82 /<br>4.22 /<br>11 F, 71 M	Plasma sialic acid<br>Anti-GM1 antibody	Colorimetric analysis for sialic acid (commercial kit); ELISA for anti-GM1 antibody	The level of plasma sialic acid was significantly lower in ASD	ASD children had higher positive rates of anti-GM1 antibodies than controls; however, there was no correlation between autistic severity and the levels of sialic acid	[45]

Blood plasma/ serum	100 / 6.4 / 44 F, 56 M	100 / 6.5 / 38 F, 62 M	Plasma sialic acid<br>Anti-GM1 antibody	Colorimetric analysis for sialic acid (periodate thiobarbituric acid method); ELISA for anti-GM1 antibody	Children with ASD had significantly higher levels sialic acid	Children with ASD had significantly higher levels anti-GM1 antibodies than healthy controls; both sialic acid and anti-GM1 antibodies levels were significantly correlated to the severity of ASD symptoms	[46]
	No control subjects	2001 / 8.94 / 260 F, 1741 M<br>A multisite cohort (12 clinical and university sites)	Global metabolome (global metabolomics profiling)	The Metabolon ultrahigh-performance liquid chromatography coupled to tandem mass spectrometry (UPLC-MS/MS); hierarchical clustering on 40 phenotypes spanning four ASD clinical domains was performed, resulting in three subgroups** with distinct phenotype patterns	Subgroup 1 shows global decreases in lipid metabolites; subgroup 2 metabolome profiles demonstrate aberrant metabolism of membrane lipids and increases in lipid oxidation products; subgroup 3 shows increases in sphingolipid metabolites and fatty acid byproducts	The study leveraged phenotypic and diagnostic information related to ASD from the Simons Simplex Collection (SSC), The Simons Foundation Autism Research Initiative (SFARI) to derive phenotypically driven subgroups and investigate their respective metabolomes	[47]
Cerebrospinal fluid	29 / 10. 4 / ? (no data)	85 / 6.9 / 20 F, 65 M	Gangliosides	Microimmunoaffinity (thin-layer chromatography, cholera toxin B (CTB) overlay analysis)	Significantly higher concentration of ganglioside GM1 in ASD	Mean ganglioside levels not different	[48]
	25 / 8.8 / ? (no data)	20 / 9 / 8 F, 12 M	Gangliosides	Microimmunoaffinity (thin-layer chromatography, cholera toxin B (CTB) overlay analysis)	Gangliosides GM1, GD1a, GD1b and GT1b were significantly increased in patients with autism	In addition to ASD, the gangliosides were determined in children with different forms of non-progressive neurological disorders (NPND) lacking	[49]

						clinical features of ASD; the reference group of patients with NPND showed significantly lower ganglioside values than the control and autistic groups	
Postmortem prefrontal cortex transcriptome data	31 / 19.7 / 7 F, 24 M	29 / 19.7 / 6 F, 23 M	Whole transcriptome data [50,51]	Metabolic modeling simulations; ASD-specific prefrontal cortex genome-scale metabolic model (GEM) using transcriptomics data; the healthy and ASD-specific models were compared via uniform sampling to identify ASD exclusive metabolic features	Several differences in the sphingolipid metabolism: higher fluxes of sphingolipid metabolism in ASD, more ceramide produced in ASD but also more ceramide converted to other derivatives, e.g. sphingomyelin or sphingosine, glucosylceramides higher in ASD	Data acquired the Gene Expression Omnibus database (ID GSE28475); controls and ASD subjects aged between 2 and 56 years; since the gene-expression levels are dependent on age, the authors chose to use the median expressions for the samples aged between 2 and 14 (not clear which of the samples were in the final study form the original 60 samples)	[52]

The terms autism, ASD, autistic disorder etc. are used as in the corresponding references. Age is given in years. ASD = autism spectrum disorder; CON = healthy controls; PDD = pervasive developmental disorder; PDD-NOS = pervasive developmental disorder – not otherwise specified; DSM = diagnostic and statistical manual of mental disorders; ADOS = autism diagnostic observation schedule; ADI-R = autism diagnostic interview-revised; L BS = broad spectrum; NQA = not quite autism.

*metabolites regarding glycosphingolipids/cholesterol, even though the studies may have included a wider array of analyses

** Subgroup 1, children with the least maladaptive behavioral traits (N = 862); subgroup 2, children with the highest degree of challenges across all phenotype domains (N = 631); subgroup 3, children with maladaptive behaviors and co-occurring conditions that showed the highest IQ scores (N= 508).

The reference (ref.) numbers correspond to the references in the main text.

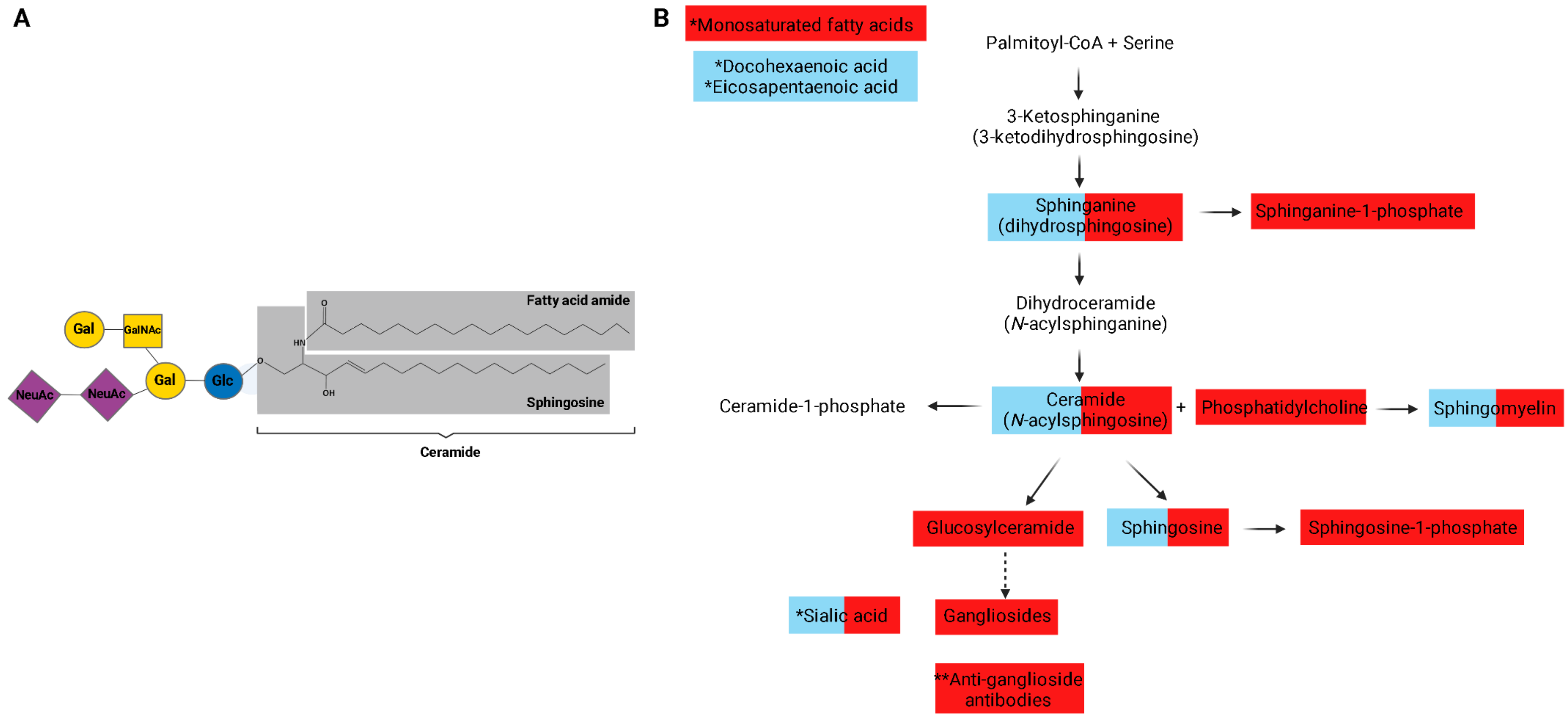

Supplementary Figure 1. A. Schematic representation of ganglioside GD1b. The glycan moiety of the ganglioside is in glycosidic linkage to the ceramide lipid which is comprised of long-chain sphingosine base bearing a fatty acid amide. Glc = glucose; Gal = galactose; GalNAc = *N*-acetylgalactose; NeuAc = sialic acid (*N*-acetylneuraminic acid). **B.** Simplified overview of sphingolipid synthesis. The color code corresponds to Table 1 in the main text where red (↑) marks elevated content in ASD compared to controls, while blue (↓) marks decreased content in ASD compared to controls. If some studies report an elevation, while others report decrease in ASD, the metabolite is shaded with both colors. *Monosaturated fatty acids, docohexaenoic and eicosapentaenoic acid, which can be found as fatty acids in the composition of ceramide (and consequently ceramide derivatives) are also included in the Figure, as well as sialic acid found in the ganglioside glycan chain. **High levels of anti-ganglioside antibodies are also reported in several studies and may correlate to higher reported ganglioside levels.

References

41. Ghezzo, A.; Visconti, P.; Abruzzo, P.M.; Bolotta, A.; Ferreri, C.; Gobbi, G.; Malisardi, G.; Manfredini, S.; Marini, M.; Nanetti, L.; et al. Oxidative Stress and Erythrocyte Membrane Alterations in Children with Autism: Correlation with Clinical Features. *PLoS One* **2013**, *8*, e66418, doi:10.1371/journal.pone.0066418.

42. Tierney, E.; Bukelis, I.; Thompson, R.E.; Ahmed, K.; Aneja, A.; Kratz, L.; Kelley, R.I. Abnormalities of Cholesterol Metabolism in Autism Spectrum Disorders. *Am. J. Med. Genet. B Neuropsychiatr. Genet.* **2006**, *141B*, 666–668, doi:10.1002/ajmg.b.30368.

43. Benachenhou, S.; Etcheverry, A.; Galarneau, L.; Dubé, J.; Çaku, A. Implication of Hypocholesterolemia in Autism Spectrum Disorder and Its Associated Comorbidities: A Retrospective Case-Control Study. *Autism Res.* **2019**, *12*, 1860–1869, doi:10.1002/aur.2183.

44. Wang, H.; Liang, S.; Wang, M.; Gao, J.; Sun, C.; Wang, J.; Xia, W.; Wu, S.; Sumner, S.J.; Zhang, F.; et al. Potential Serum Biomarkers from a Metabolomics Study of Autism. *J. Psychiatry Neurosci.* **2016**, *41*, 27–37, doi:10.1503/jpn.140009.

45. Yang, X.; Liang, S.; Wang, L.; Han, P.; Jiang, X.; Wang, J.; Hao, Y.; Wu, L. Sialic Acid and Anti-Ganglioside Antibody Levels in Children with Autism Spectrum Disorders. *Brain Res.* **2018**, *1678*, 273–277, doi:10.1016/j.brainres.2017.10.027.

46. Ashaat, E.A.; Sabry, S.; Zaki, M.E.; Mohamed, R.; Abdelsattar, H.A.; Bawady, S.A.; Ashaat, N.A.; Elnaggar, W.; Ganem, M.M.F.; El-Hariri, H.M.; et al. Sialic Acid and Anti-Ganglioside M1 Antibodies Are Invaluable Biomarkers Correlated with the Severity of Autism Spectrum Disorder. *Brain Dev.* **2023**, *45*, 212–219, doi:10.1016/j.braindev.2022.11.006.

47. Prince, N.; Chu, S.H.; Chen, Y.; Mendez, K.M.; Hanson, E.; Green-Snyder, L.; Brooks, E.; Korrick, S.; Lasky-Su, J.A.; Kelly, R.S. Phenotypically Driven Subgroups of ASD Display Distinct Metabolomic Profiles. *Brain Behav. Immun.* **2023**, *111*, 21–29, doi:10.1016/j.bbi.2023.03.026.

48. Nordin, V.; Lekman, A.; Johansson, M.; Fredman, P.; Gillberg, C. Gangliosides in Cerebrospinal Fluid in Children with Autism Spectrum Disorders. *Dev. Med. Child Neurol.* **1998**, *40*, 587–594, doi:10.1111/j.1469-8749.1998.tb15423.x.

49. Lekman, A.; Skjeldal, O.; Sponheim, E.; Svennerholm, L. Gangliosides in Children with Autism. *Acta Paediatr.* **1995**, *84*, 787–790, doi:10.1111/j.1651-2227.1995.tb13757.x.

50. Chow, M.L.; Li, H.-R.; Winn, M.E.; April, C.; Barnes, C.C.; Wynshaw-Boris, A.; Fan, J.-B.; Fu, X.-D.; Courchesne, E.; Schork, N.J. Genome-Wide Expression Assay Comparison across Frozen and Fixed Postmortem Brain Tissue Samples. *BMC Genomics* **2011**, *12*, 449, doi:10.1186/1471-2164-12-449.

51. Chow, M.L.; Winn, M.E.; Li, H.-R.; April, C.; Wynshaw-Boris, A.; Fan, J.-B.; Fu, X.-D.; Courchesne, E.; Schork, N.J. Preprocessing and Quality Control Strategies for Illumina DASL Assay-Based Brain Gene Expression Studies with Semi-Degraded Samples. *Front. Genet.* **2012**, *3*, 11, doi:10.3389/fgene.2012.00011.

52. Esvap, E.; Ulgen, K.O. Neuroinflammation, Energy and Sphingolipid Metabolism Biomarkers Are Revealed by Metabolic Modeling of Autistic Brains. *Biomedicines* **2023**, *11*, 583, doi:10.3390/biomedicines11020583.
